# Evaluation of PET performance and MR compatibility of a preclinical PET/MR insert with digital silicon photomultiplier technology

**DOI:** 10.1186/2197-7364-2-S1-A55

**Published:** 2015-05-18

**Authors:** Patrick Hallen, David Schug, Jakob Wehner, Bjorn Weissler, Pierre Gebhardt, Benjamin Goldschmidt, Andre Salomon, Peter Duppenbecker, Fabian Kiessling, Volkmar Schultz

**Affiliations:** Department of Physics of Molecular Imaging Systems, RWTH Aachen University, Germany; Department of Chemical Application Research, Philips Research, Germany; Division of Imaging Sciences and Biomedical Engineering, King’s College London, UK; Department of Oncology Solutions, Philips Research, Germany; Institute for Experimental Molecular Imaging, RWTH Aachen University, Germany

In this work we present detailed characterizations of our preclinical high resolution PET/MR insert based on the Hyperion-IID platform. The PET/MR insert consists of a ring of 10 singles detection modules, each comprising 2x3 scintillation detector stacks. Each detector stack features a 30x30 pixelated LYSO crystal array with a height of 12 mm and a pitch of 1 mm, coupled via a slit 2 mm light guide to a digital SiPM tile. The PET performance is stable under a wide range of operating points. The spatial resolution is below 1Ä,mm and the CRT reaches 260 or 450 ps depending on trigger settings. The energy resolution is 12.6% FWHM. The characterization of the MR compatibility showed no relevant degradation in PET performance during MRI operation. On the MRI side, we observe a degradation in B0 homogeneity from a VRMS of 0.03 ppm to 0.08 ppm with active shimming, while observing only minor degradations in the B0 field. The noise floor is slightly increased by 2-15% without any observable dependence on the activity. The Z gradients induces an observable eddy current inside the PET inserts which can lead to ghosting artifacts for EPI sequences. However, we don't observe any visible image degradation for widely used anatomical imaging sequences such as gradient echo and turbo spin echo sequences. To prove the viability of our PET/MR insert for in vivo small animal studies, we successfully performed a longitudinal mouse study with subcutaneously injected tumor model cells. The simultaneously acquired PET/MR images provide a high level of anatomical information and soft tissue contrast in the MR layer together with a high resolution image of the FDG tracer distribution in the PET layer.

